# Reduced mitochondrial respiration in peripheral T cells after paediatric heamatopoietic stem cell transplantation

**DOI:** 10.3389/fimmu.2023.1327977

**Published:** 2024-01-10

**Authors:** Kasper Mølgaard, Katrine Kielsen, Marianne Ifversen, Özcan Met, Inge Marie Svane, Klaus Müller

**Affiliations:** ^1^ National Center for Cancer Immune Therapy, Department of Oncology, Herlev Hospital, Copenhagen, Denmark; ^2^ Institute of Inflammatory Research, Rigshospitalet, Copenhagen, Denmark; ^3^ Hematopietic Stem Cell Transplantation and Primary Immune Deficiency, Department of Paediatric and Adolescent Medicine, Copenhagen University Hospital Rigshospitalet, Copenhagen, Denmark; ^4^ Institute of Clinical Medicine, University of Copenhagen, Copenhagen, Denmark

**Keywords:** hematopoietic stem cell transplantation, acute graft-versus-host disease, mitochondrial fitness, spare respiratory capacity, real-time metabolism, T cells

## Abstract

**Background:**

Recovery and functional differentiation of T-cell subsets are central for the development of immune function and complications after allogeneic hematopoietic stem cell transplantation (HSCT), but little is known about the cellular respiration and factors influencing T-cell metabolic fitness during immune maturation after HSCT.

**Method:**

We included 20 HSCT patients and analysed mitochondrial oxidative phosphorylation and mitochondrial fitness in peripheral blood mononuclear cell samples collected at days +90 and +180 after HSCT.

**Results:**

Phenotypic analysis revealed lower overall T-cell counts, lower CD4+/CD8+ ratio and a skewed distribution of early T-cell subsets at day +90, gradually recovering by day +180. Although ATP turnover in HSCT patients was similar to healthy controls, the spare respiratory capacity (SRC) of T cells, reflecting the available energy reserve, was significantly reduced at day +90 and +180 compared to healthy controls. This reduction in SRC was not correlated with the occurrence of acute graft-versus-host disease (aGVHD), the intensity of conditioning regimens and markers of T-cell exhaustion.

**Conclusion:**

We found significantly depressed SRC until six months post-HSCT, but we were not able to identify transplant-related risk factors or associations with the clinical outcome.

## 1 Introduction

A well-balanced reconstitution of T cells is essential for the immune recovery following allogeneic hematopoietic stem cell transplantation (HSCT) to limit side effects such as acute graft-versus-host disease (aGVHD) and infections while maintaining the T-cell mediated graft-versus-leukaemia (GvL) effect (1, 2). In HSCT, T-cell recovery is based on a peripheral expansion of mature donor T cells and the *de novo* generation of T cells in the thymus from donor-derived progenitor cells. As thymic T-cell production takes several months to occur, the peripheral homeostatic expansion of T cells is essential to maintain protective immune function towards infections (3).

The homeostatic cytokines, IL-7 and IL-15, are essential for the early expansion of memory T cells giving rise to long-term immunity and protection, as well as the development of T cells expressing CD45RA and the lymph node-homing antigen CCR7 after both HSCT and adoptive T-cell therapies (ACT) (4–7). Our group has previously shown that plasma levels of IL-7 peak at the time of maximum lymphopenia and that increased IL-7 levels are associated with increased risk of grade II-IV aGVHD and reduced overall survival after HSCT (8). In addition, we and others have presented evidence that metabolic profiling of T cells using mitochondrial respiration may be useful as a marker for long-term T-cell persistence in immunotherapeutic settings (9, 10).

The mitochondrial or metabolic T cell fitness is often analysed as the oxygen consumption rate (OCR), measured as the rate of oxygen consumed in the mitochondria of T cells at their baseline conditions (basal respiration) and after manipulation to determine the maximal rate of oxygen (maximal respiration), the oxygen consumption is directly linked to oxidative phosphorylation (ATP turnover) and the theoretical extra capacity within a T cell to produce ATP as a response to increased energetic demands (the spare respiratory capacity, SRC). We recently showed that cytokine-directed differentiation of T cells using IL-2 and IL-15 could be distinguished using metabolic profiling (11). These metabolic profiles differ in SRC, and others have also described how mitochondrial respiration influences T-cell proliferation, differentiation, and persistence in ATC. Accordingly, analysing SRC by real-time metabolic analysis can be a useful tool in determining T-cell fate (4, 9, 11, 12). Cytokines like IL-7 and IL-15, which were found to be essential in determining the outcome of HSCT, appear to be crucial for the generation of a persistent T cell phenotype with self-renewing properties (6) which is associated with a defined metabolic fitness, utilizing oxidative phosphorylation instead of glycolysis and having an increased SRC (13–15). Furthermore, several mechanisms are capable of reverting persistence and reactivity in T cells, including manipulation of cytokines and stimulation (16, 17). To our knowledge, the metabolic fitness of T cells during immune reconstitution following HSCT and their association with transplant-related variables and complications such as aGVHD has not yet been investigated.

In this study, we investigated the metabolic respiration in T cells from children undergoing HSCT, and how metabolic respiration associates with T cell phenotypes of naïve-, memory-, effector memory-, and terminal effector differentiation, and metabolicfitness. We demonstrate that metabolic traits in standard parameters of basal respiration and ATP production in HSCT patients are similar to healthy young adults, while SRC is significantly impaired after HSCT.

## 2 Materials and methods

### 2.1 Patient population

Patient samples were collected from children undergoing allogenic HSCT between 2015 and 2020 at Copenhagen University Hospital Rigshospitalet, Denmark. For this study, we included 20 patients (age 9.6 years, 3-16 years) with available cryo-preserved mononuclear cells from days +90 and +180 post-transplant. Other inclusion criteria were transplantation with myeloablative conditioning, a bone marrow graft, matched sibling, or unrelated donor, and either absence of aGVHD and no reactivation of cytomegalovirus or Epstein-Barr virus activation post-transplant (n=11) or grade II-IV aGVHD (n=9) (**Table 1**).

**Table 1:** Overview of HSCT patient characteristics.

Patient cohort (n=20)	Day +90	Day +180
**Included samples, n (%)**	19 (95%)	20 (100%)
**Age in years, mean (range)**	10.1 (3.1-16.1)	9.6 (3.1-15.9)
Sex, n (%)		
Male	11 (55%)	11 (55%)
Female	8 (40%)	9 (45%)
Disease, n (%)		
AML	1 (5%)	1 (5%)
ALL	5 (15%)	5 (25%)
MDS	3 (15%)	4 (20%)
Sickle cell anaemia	4 (20%)	4 (20%)
Other anaemia	4 (20%)	4 (20%)
Primary immunodeficiencies	2 (10%)	2 (10%)
Myeloablative conditioning, n (%)		
High intensity	13 (65%)	14 (70%)
TBI + VP16	2 (10%)	2 (10%)
BU + CY ± MEL	3 (15%)	4 (20%)
THIO + FLU + BU/TREO	8 (45%)	8 (40%)
Reduced intensity	6 (30%)	6 (30%)
TREO + FLU	3 (15%)	3 (15%)
CY ± FLU	3 (15%)	3 (15%)
**Anti-thymocyte-globulin, n (%)**	**14 (70%)**	**14 (70%)**
Donor type, n (%)		
Matched sibling donor (10/10)	9 (45%)	10 (50%)
Matched unrelated donor (9/10 or 10/10)	10 (50%)	10 (50%)
GvHD at maximum, n (%)		
Non (0)	11 (55%)	11 (55%)
Mild (I)	0 (0%)	0 (0%)
Moderate (II)	7 (35%)	7 (35%)
Severe (III-IV)	1 (5%)	2 (10%)
Viral reactivation		
Cytomegalovirus	3 (15%)	3 (15%)

Included patients from the original cohort with available samples for Seahorse analyses at days +90 and +180. Percentages refer to the original cohort. AML, acute myeloid leukaemia; ALL, acute lymphoblastic leukaemia; MDS, myelodysplastic disorder; TBI, Total Body Irradiation; VP16, Etoposide; BU, Busulfan; CY, Cyclophosphamide; MEL, Melphalan; THIO, Thiotepa; FLU, Fludarabine; TREO, Treosulfan.

The study protocol was approved by the Ethics Committees of the Capital Region of Denmark (#H-7-2014-016) and written informed consent was obtained from all patients and/or their legal guardians.

### 2.2 Healthy controls

Blood samples from 11 healthy controls with a mean age of 24.13 years (aged 23-26) were healthy blood donors attending the Blood Bank at Copenhagen University Hospital Rigshospitalet. These donors were anonymous to the investigators, and thus no ethical approval was required according to Danish legislation.

### 2.3 Peripheral blood collection and cell isolation

Blood samples were obtained from patients at day +90 and day +180 post-HSCT. Peripheral blood mononuclear cells (PBMCs) were isolated by gradient centrifugation of heparinized blood using Lymphoprep (Axis-Shield, Scotland), washed three times in PBS and resuspended in RPMI 1640 (Lonza, Switzerland) containing 30% bovine serum (Biological Industries, Israel) and 10% Dimethyl Sulfoxide (VWR, United states). Then PBMCs were aliquoted into portions of 5.0x10^6^ cells per mL, cryo-preserved and stored in liquid nitrogen until analysis.

### 2.4 Human primary cultures

PBMCs were thawed using X-VIVO 15 media (LONZA, Switzerland) supplemented with 5% human serum (Gibco) and 200 U/mL IL-7 and IL-15 (PeproTech, United States) and washed once in media before being cultured. Cells were cultured in 24-well tissue culture plates (NUNC, Denmark) and maintained at concentrations of 1x10^6^ cells/mL.

### 2.5 Real-time metabolic profiling

Measurements of oxygen consumption rate (OCR) were performed on a Seahorse XFe96 analyser (Agilent Technologies, United States) as previously described (11). Briefly, on day 0 XF RPMI media (Agilent Technologies, United States) were pre-warmed in a non-CO_2_ regulated incubator, and a sensor cartridge plate (Agilent Technologies, United States) was prepared by hydrating sensor probes in 200 μL XF calibrant solution (Agilent Technologies, United States) overnight. On day 1, XF RPMI media would be prepared with 1 mM pyruvate (Agilent Technologies, United States), 3 mM L-glutamine (Agilent Technologies) and 4 mM glucose (Agilent Technologies). Oligomycin (Sigma-Aldrich), antimycin A (Sigma-Aldrich, United States) and carbonyl cyanide-p trifluoromethoxy phenylhydrazone (FCCP) (Sigma-Aldrich, United States) were prepared in supplemented XF RPMI media. 20 μL 10x oligomycin was loaded to the left half side of the cartridge plate in port A and 20 μL 10x FCCP was loaded to the right half side in port A. Antimycin A was loaded to every single port C in the cartridge plate. In optimization runs a titration of cells would be made on both the left and right side of the cartridge plate and inhibitor titrations would be performed using ports A, B, C and D on the left side (oligomycin) and the right side (FCCP). Additional unused ports were filled with XF media as injections are done by air pressure, and empty wells disrupt the build-up of pressure and injection efficiency.

For cell preparations, an XF cell culture plate (Agilent Technologies, United States) was coated with a 0.25 mg/mL Cell-Tak (Corning, United States) in sodium carbonate (NaHCO_3_) (Sigma-Aldrich, United States) and sodium hydroxide (NaOH) (Sigma-Aldrich, United States) solution. 10-12 μL were added to each well and coated for 30 minutes at room temperature. After 30 minutes, the Cell-Tak solution was removed and washed once with 200 μL sterile water and with 200 μL sterile PBS, before being left to dry at room temperature for at least 30 minutes. HSCT patient and healthy control samples were harvested and counted on an automated cell counter. Cells were resuspended to 400.000 cells per 180 μL and transferred to a coated and dry XF cell plate. The cell plate was centrifuged for 10 minutes at 1000 xg to bind non-adherent T cells. The cell plate was left to pre-warm in a non-CO_2_-regulated incubator for at least 30 minutes.

Basal respiration was calculated as the difference in OCR from the last measurement before injection and the second measurement after injection of antimycin A. Maximal respiration was calculated as the difference in OCR from the first measurement after treatment with FCCP and the second measurement after injection of antimycin A. ATP turnover was calculated as the difference in baseline respiration and after the addition of oligomycin (second measurement) (**Supplementary Figure 2**, **Supplementary Data 1**).

### 2.6 Phenotypic analysis using flow cytometry

Phenotypic analysis of T cells using flow cytometry was done by staining samples in 96-well plates using around 2-300.000 cells per staining. Samples were stained with a reference staining of anti-human CD3 (BV786, clone UCHT1, BD Biosciences, United States), anti-human CD4 (BV510, clone SK3, BD Biosciences, United States), anti-human CD8 (PECF594, clone HIT8a, BD Biosciences, United States) and a dead cell marker (LIVE/DEAD Nir, Thermo Fisher, United States). The reference specimen was used to determine basic phenotypes and to set gates for additional markers stained in the sample specimen. All panels were standard panels used at our facilities and were compensated using single colour staining and checked with fluorescence minus one (FMO) controls.

Phenotypic analyses were done using a panel consisting of three sample specimens. Reference gating was used to investigate positive populations in specimen samples of T-cell differentiation (Tdiff), and T-cell exhaustion (Tex) and to analyse additional populations in samples (Tother). In-depth phenotyping was performed similarly to previously described, where Tdiff samples were washed once and stained with antibodies: anti-human γδ (BV421, clone B1, BD Biosciences, United States), anti-human CD4 (BV510, clone SK3, BD Biosciences, United States), anti-human CD28 (BV605, clone CD28.2, BD Biosciences, United States), anti-human CD127 (BV650, clone HIL-7R-M21, BD Biosciences, United States), anti-human CD27 (BV711, clone L128, BD Biosciences, United States), anti-human CD3 (BV786, clone UCHT1, BD Biosciences, United States), anti-human CD57 (FITC, clone NK-1, BD Biosciences, United States), anti-human HLA-DR (PerCP-Cy5.5, clone L243, BD Biosciences, United States), anti-human CCR7 (PE, clone 2-L1-A, BD Biosciences, United States), anti-human CD8 (PE-CF584, clone HIT8a, BD Biosciences, United States), anti-human PD-1 (Pe-Cy7, clone EH12.1, BD Biosciences, United States), anti-human CD45RA (APC, clone HI100, BD Biosciences, United States), anti-human CD25 (APC-R700, clone 2A3, BD Biosciences, United States) and dead cell marker (LIVE/DEAD Nir, Thermo Fisher, United States). Tex samples were stained with anti-human CD39 (BV421, clone A1, BD Biosciences, United States), anti-human CD4 (BV510, clone SK3, BD Biosciences, United States), anti-human TIM3 (BV605, clone 7D3, BD Biosciences, United States), anti-human TIGIT (BV650, Clone 1G9, BD Biosciences, United States), anti-human CD3 (BV786, clone UCHT1, BD Biosciences, United States), anti-human LAG3 (FITC, clone 11C3C65, BioLegend, United States), anti-human DNAM (PerCP-Cy5.5, clone 11A8, BioLegend, United States), anti-human CCR7 (PE, clone 3D12, BD Biosciences, United States), anti-human CD8 (PE-CF584, clone HIT8a, BD Biosciences, United States), anti-human PD-1 (Pe-Cy7, clone EH12.1, BD Biosciences, United States), anti-human CD45RA (APC, clone HI100, BD Biosciences, United States) and dead cell marker (LIVE/DEAD Nir, Thermo Fisher, United states). Tother samples were stained with anti-human NKG2a (BV421, clone 131411, BD Biosciences, United States), anti-human CD33 (BV510, clone WM53, BD Biosciences, United States), anti-human CD123 (BV605, clone 7G3, BD Biosciences, United States), anti-human CD16 (BV650, clone 3G8, BD Biosciences, United States), anti-human CD19 (BV711, clone HIB19, BD Biosciences, United States), anti-human CD3 (BV786, clone UCHT1, BD Biosciences, United States), anti-human CD56 (FITC, clone B159, BD Biosciences, United States), anti-human HLA-DR (PerCP-Cy5.5, clone L243, BD Biosciences, United States), anti-human CD11c (PE, clone BU15, BD Biosciences, United States), anti-human CD14 (PE-CF584, clone MφP9, BioLegend, United States), anti-human PD-1 (Pe-Cy7, clone EH12.1, BD Biosciences, United States), anti-human CD1c (APC, clone L161, BioLegend, United States) and dead cell marker (LIVE/DEAD Nir, Thermo Fisher, United States). PBMCs from HSCT patients were limited in some patients and thus flow cytometry analyses were priority ranked according to Tdiff, Tex and Tother. All flow cytometry data were collected on a Quanteon flow cytometer, compensated using single stained PBMCs. Analysis was carried out using FlowJo v10 software and gates were made using FMOs (**Supplementary Figure 1**).

### 2.7 Statistical analysis

For comparison of SRC in patient groups and healthy controls we used the Mann-Whitney unpaired nonparametric U-test, often used for non-normally distributed data with unequal numbers or small sample sizes (less than 20 per group). For comparison of patient data on days +90 and +180 we used the Wilcoxon signed-rank test. For the correlation of SRC on day +90 or day +180 and the CRP values, we used Spearman’s rank correlation test. All statistical analyses were done using GraphPad Prism 8. Statistically significant differences (p < 0.05) were denoted in figures as calculated p-values.

## 3 Results

### 3.1 Patient characteristics

We analysed PBMC samples from HSCT patients obtained on days +90 and +180 post-transplant. From the cohort of 20 children, we were able to obtain sufficient PBMC numbers from 19 samples (95%) at day +90 and 20 samples (100%) at day +180 (**Table 1**).

Patient characteristics are illustrated in **Table 1**. Patients were transplanted for either malignant (n=10) or benign disorders (n=10) with either an HLA-matched sibling donor (n=10) or a matched unrelated donor (n=10). Nine patients developed aGVHD grade II-IV at a median of 14 days post-transplant (range: 7-30), and of these, three patients developed CMV reactivation requiring pre-emptive treatment.

### 3.2 T-cell phenotyping after HSCT

**Figure 1:**
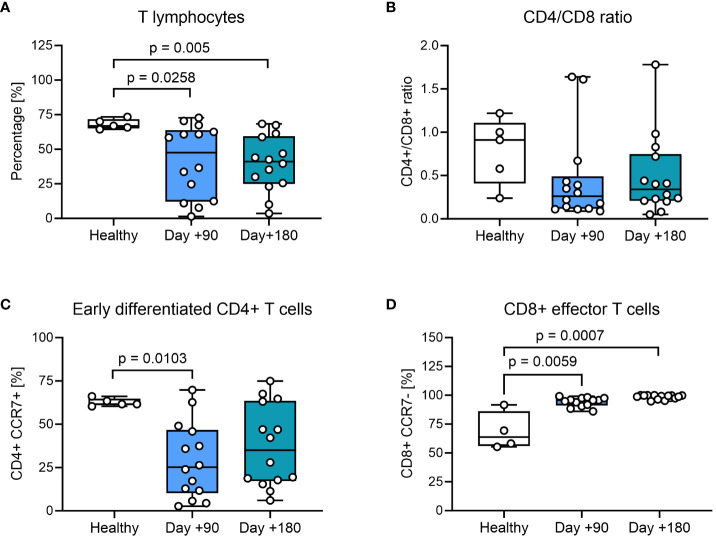
T-cell phenotyping of healthy controls and HSCT patients using flow cytometry. **(A)** Proportion of CD3+ T cells from living PBMCs. **(B)** The CD4+/CD8+ ratio within the CD3+ T-cell population. **(C)** Early differentiated CD4+ cells: CCR7+ CD4+ memory T cells present within the CD4+ T-cell population, defined as T_N_ (CD45RA+ CCR7+) and T_CM_ (CD45RA- CCR7+). **(D)** CD8+ effector T cells: CCR7- CD8+ effector T cells present within the CD8+ T-cell population, defined as T_EM_ (CD45RA- CCR7-) and T_TE_ (CD45RA+ CCR7-). The level of significance was calculated using a Mann-Whitney test.

We compared the phenotypic characteristics of PBMCs from healthy controls with HSCT patients at days +90 and +180 after transplantation. In healthy controls CD3+ T cells constituted 65-75% of PBMCs, while children undergoing HSCT had a significantly lower proportion of CD3+ T cells at day +90 and +180, confirming a prolonged T cell recovery time after HSCT with myeloablative conditioning (**Figure 1A**). We also found indications of a lower CD4+/CD8+ T-cell ratio in most HSCT patients compared to healthy controls at both days +90 and +180 (**Figure 1B**). When looking closer into the CD4+ T-cell subset, the percentage of CCR7+ memory subsets was significantly lower at day +90 compared with healthy controls and remained decreased at day +180, although not reaching statistical significance (**Figure 1C**). When addressing the differentiation of CD8+ T cells, we found significantly fewer CCR7- effector cells at day +90 and at day +180 after HSCT compared to healthy controls (**Figure 1D**).

### 3.3 Mitochondrial respiration in T cells post-transplantation

**Figure 2:**
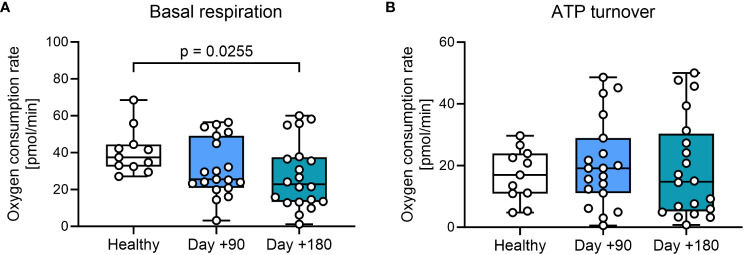
Mitochondrial respiration in T cells from HSCT patients and healthy controls. **(A)** Mitochondrial basal respiration measured before treatment with mitochondrial modulators. **(B)** ATP-linked mitochondrial respiration measured after the inhibition of the ATPase using 2.0 μM oligomycin. The level of significance was calculated using a Mann-Whitney test.

We then investigated mitochondrial respiration in PBMCs. First, we evaluated standard markers for metabolic fitness, basal respiration, and ATP turnover in the total patient cohort. We found a minor, but significant decrease in basal respiration in PBMCs at day +180 after HSCT compared with healthy controls (**Figure 2A**, **Supplementary Table 1**). Next oxygen consumption directly related to ATP turnover was studied by treating samples with oligomycin, with findings of comparable ATP-linked mitochondrial respiration in patient samples after HSCT compared with healthy controls (**Figure 2B**).

**Figure 3:**
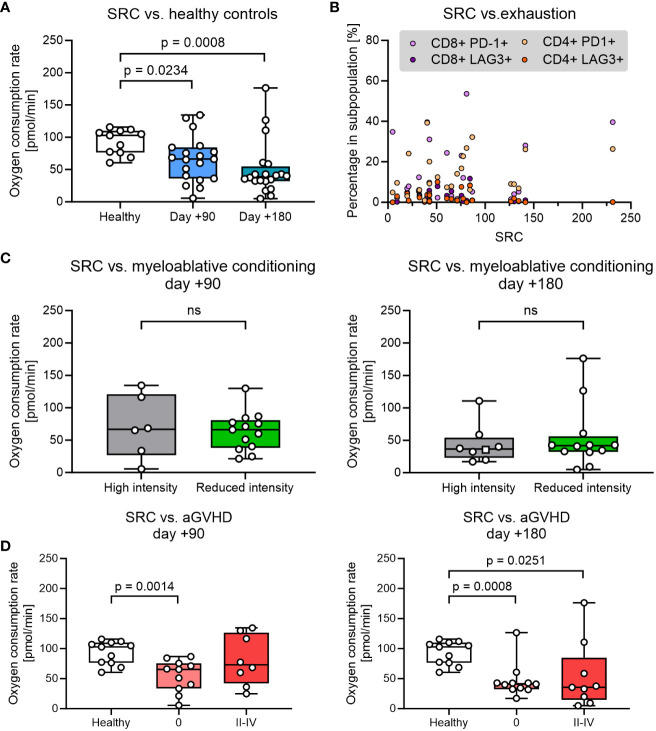
Analysis of spare respiratory capacity (SRC) was analysed over different parameters. **(A)** SRC at days +90 and +180 after pediatric HSCT compared with healthy control. **(B)** Proportion of exhausted T cells (defined by expression of LAG3 and PD-1) as a function of SCR. **(C)** The influence of the myeloablative conditioning regimen on SRC. **(D)** The influence of aGVHD on SRC. The level of significance was calculated using a Mann-Whitney test or Spearman’s rank correlation coefficient.

The spare respiratory capacity (SRC) is an effective marker to quantify the potential of T cells regarding memory T cell development, cytotoxic capacity, and long-term persistence in circulation. We measured the theoretical maximal- and basal oxygen consumption (OCR) in PBMCs by manipulating the mitochondrial membrane with the uncoupler carbonyl cyanide-p trifluoromethoxy phenylhydrazone (FCCP) and oligomycin and used this for estimating SRC (**Supplementary Figure 2**, **Supplementary Table 1**). Compared with healthy controls we found a significantly lower SRC in patient cells obtained at day +90 after HSCT, which further decreased at day +180 (**Figure 3A**). T-cell exhaustion measured by expression of LAG3 and PD-1 did not correlate significantly with SRC in either CD4+ or CD8+ T cells from HSCT patients (p>0.05) (**Figure 3B**).

Next, we investigated the impact of clinical parameters that could be potentially important for the reduction of SRC. SRC in PBMCs was found to be comparable in HSCT patients undergoing high intensity (TBA+VP16, BU+CY+MEL or THIO+FLU+TREO) and low-intensity conditioning (TREO+FLU or CY+FLU) at both days +90 and +180 (**Figure 3C**). Likewise, decreased SRC at days +90 and +180 was not associated with age, donor type (sibling vs. unrelated donor), disease (benign vs. malignant) or anti-thymocyte globulin (ATG) administration (p>0.05, data not shown).

Finally, the SRC in HSCT patients were then compared between patients without aGVHD (grade 0) and grade II-IV aGVHD requiring immunosuppressive treatment with steroids. SRC was found to be reduced in HSCT patients irrespective of aGvHD status at both time points (although not reaching significance for patients with aGVHD at day +90) and with no significant difference in SRC between patients with no aGVHD and grade II-IV aGVHD at any time point (**Figure 3D**).

We investigated if SRC in PBMCs from HSCT patients was affected by the degree of systemic inflammation early post-transplant. No significant correlation could be obtained between the maximum circulating CRP levels and SRC at day +90 or +180. Likewise, accumulated doses of steroids within the first 100 days, did not correlate with SRC at any of the time points (data not shown).

## 4 Discussion

We have previously described that mitochondrial fitness and especially spare respiratory capacity, SRC, can be markers for T-cell proliferation, persistence in circulation, reactivity, and T-cell phenotype *in vitro* and eventually in patients receiving T-cell therapies (11, 12). Others have demonstrated that donor T cells persisting in patients after HSCT have specific signatures reflecting T memory subsets, classified by specific metabolic phenotypes and metabolic fitness (4, 9). Such metabolic features have also been shown to be important for tumour control where increased T cell glycolysis, and reduced oxidative phosphorylation as well as lowered SRC, were shown to inhibit T cell antitumour immunity (18).

In this study, we investigated the mitochondrial markers of basal respiration, ATP turnover and SRC in T cells from PBMC samples and how these associate with T cell recovery at days +90 and +180 after transplantation where the early reconstitution of T cells is mainly driven by the peripheral expansion of mature donor T cells and confirmed by an increased in the CD8+ CCR7- population (3). Such an expansion is highly ATP dependent and therefore we compared the ATP-driven mitochondrial respiration between patients and healthy controls. We observed no difference in ATP turnover, but basal respiration at day +180 after HSCT showed a small, but significant drop in oxygen consumption rates, and a clear decrease in SRC at days +90 and +180 indicated reduced metabolic fitness in the patients. This suggests that increased energy demands during peripheral expansion of T cells post-HSCT do not seem to be met by increased mitochondrial oxidative phosphorylation but could result from other metabolic pathways such as aerobic glycolysis, that has previously been associated with T-cell proliferation, effector function, and cytokine production (19–21). A rewiring of T cell metabolism could also be in line with the findings of decreased SRC as exhausted mitochondrial respiration would lead to a shift into alternative metabolic pathways, whereas thymic T cell expansion would result in a naïve population with increased SRC (3, 9, 22, 23).

Previous studies investigating the metabolic direction of T cells under PD-1 engagement have shown that PD-1 interaction blocks the ability to engage in glycolysis and seems to play a role in the restoration of SRC (24–26). PD-1 expression on CD4+ and CD8+ has been reported to be high early after HSCT but significantly decreasing after 100 days post-transplant (27), which is consistent with our findings of low expression of PD-1 and LAG-3 and undetectable levels of TIM-3 and TIGIT markers at day +90 and +180 after HSCT. Consequently, downregulation of PD-1 and reduced regulation of T-cell function after peripheral activation could drive an increased glycolytic metabolism in T cells and thus explain a reduced SRC at days +90 and +180, but we were unable to detect any significant association between the PD-1 expression and reduction in SRC.

Conditioning regimens with highly intensive chemotherapy and total body irradiation are commonly used for conditioning in HSCT but are known to carry an increased risk of complications, such as aGVHD, compared with less toxic regimens (28). Our data showed no significant difference in SRC across conditioning regimens at both days +90 and +180, indicating that the intensity of the conditioning does not influence mitochondrial respiration in donor-derived T cells after transplantation. Likewise, the level of systemic inflammation was not significantly related to a reduction in SRC. As the burden of morbidity and cytotoxic treatment given before referral to HSCT could be a confounding factor we investigated the impact of malign/benign diagnosis. However, diagnosis did not appear to affect SRC at days +90 and +180 as we observed similar degrees of decreases in SRC at these timepoints regardless of malign/benign diagnosis. We could however speculate that the presence of tumour specific T cells in peripheral blood could have an impact on SRC. Such studies would benefit from patient samples prior to HSCT, which were, however, not available for this study.

We expected lower SRC in PBMCs from patients diagnosed with aGVHD as a sign of T-cell exhaustion following the activation of alloreactive T cells. Although patients with aGVHD grade II-IV showed a reduction in SRC at day +180 after HSCT compared to healthy controls there was no significant difference when comparing patients with and without aGvHD.

This study is limited by the rather small and heterogenous study population and therefore associations between the spare respiratory capacity and clinical variables such as conditioning regimens and aGVHD status could not be fully explored. Future investigations with larger and more homogenous cohorts are needed to adequately address these associations as well as non-aGvHD related immune reactions.

This study is also limited by the timing of sampling, and further studies in earlier obtained samples would be of great interest although low numbers of T cells at earlier timepoints could be a challenge. Further, a more comprehensive understanding of low mitochondrial fitness in HSCT outcomes may be revealed by analysing the donor graft before infusion as well as patient samples prior to treatment.

Finally, our control group consisting of young adults was older than the patient group, as blood sampling from healthy children was omitted for ethical reasons. However, we would not expect any significant age-related difference mitochondrial respiration in peripheral blood T cells between young adults compared to our patient cohort.

In conclusion, we have presented the first preliminary evidence strongly suggesting a significant dysregulation in the metabolic profiles of patients after HSCT. Future studies involving a larger cohort are necessary to further explore risk factors as well as the potential impact on the clinical outcomes at both shorter and longer term.

## Data availability statement

The raw data supporting the conclusions of this article will be made available by the authors, without undue reservation.

## Ethics statement

The studies involving humans were approved by Ethics Committees of the Capital Region of Denmark (#H-7-2014-016). The studies were conducted in accordance with the local legislation and institutional requirements. The human samples used in this study were acquired from primarily isolated as part of your previous study for which ethical approval was obtained. Written informed consent for participation was not required from the participants or the participants’ legal guardians/next of kin in accordance with the national legislation and institutional requirements.

## Author contributions

KJ: Conceptualization, Data curation, Formal Analysis, Investigation, Methodology, Project administration, Resources, Software, Supervision, Validation, Visualization, Writing – original draft, Writing – review & editing. KK: Conceptualization, Data curation, Formal Analysis, Methodology, Project administration, Supervision, Validation, Visualization, Writing – original draft, Writing – review & editing. MI: Funding acquisition, Writing – review & editing. ÖM: Funding acquisition, Project administration, Supervision, Writing – review & editing. IS: Funding acquisition, Supervision, Writing – review & editing. KM: Conceptualization, Formal Analysis, Funding acquisition, Project administration, Supervision, Writing – original draft, Writing – review & editing.
