# Supplementary material for: Reduced mitochondrial respiration in peripheral T cells after paediatric heamatopoietic stem cell transplantation
Source: Front Immunol. 2024 Jan 10;14:1327977. doi: 10.3389/fimmu.2023.1327977 (PMC10806108; doi:10.3389/fimmu.2023.1327977)
Supplement: Supplementary file 1 [file DataSheet_1.docx]

Supplementary Material

# Supplementary Data

Supplementary Material should be uploaded separately on submission. Please include any supplementary data, figures and/or tables.

Supplementary material is not typeset so please ensure that all information is clearly presented, the appropriate caption is included in the file and not in the manuscript, and that the style conforms to the rest of the article.

# Supplementary Figures and Tables

For more information on Supplementary Material and for details on the different file types accepted, please see [here](https://www.frontiersin.org/guidelines/author-guidelines#supplementary-material).

## Supplementary Figures

### Supplementary table

### Supplementary figure

| 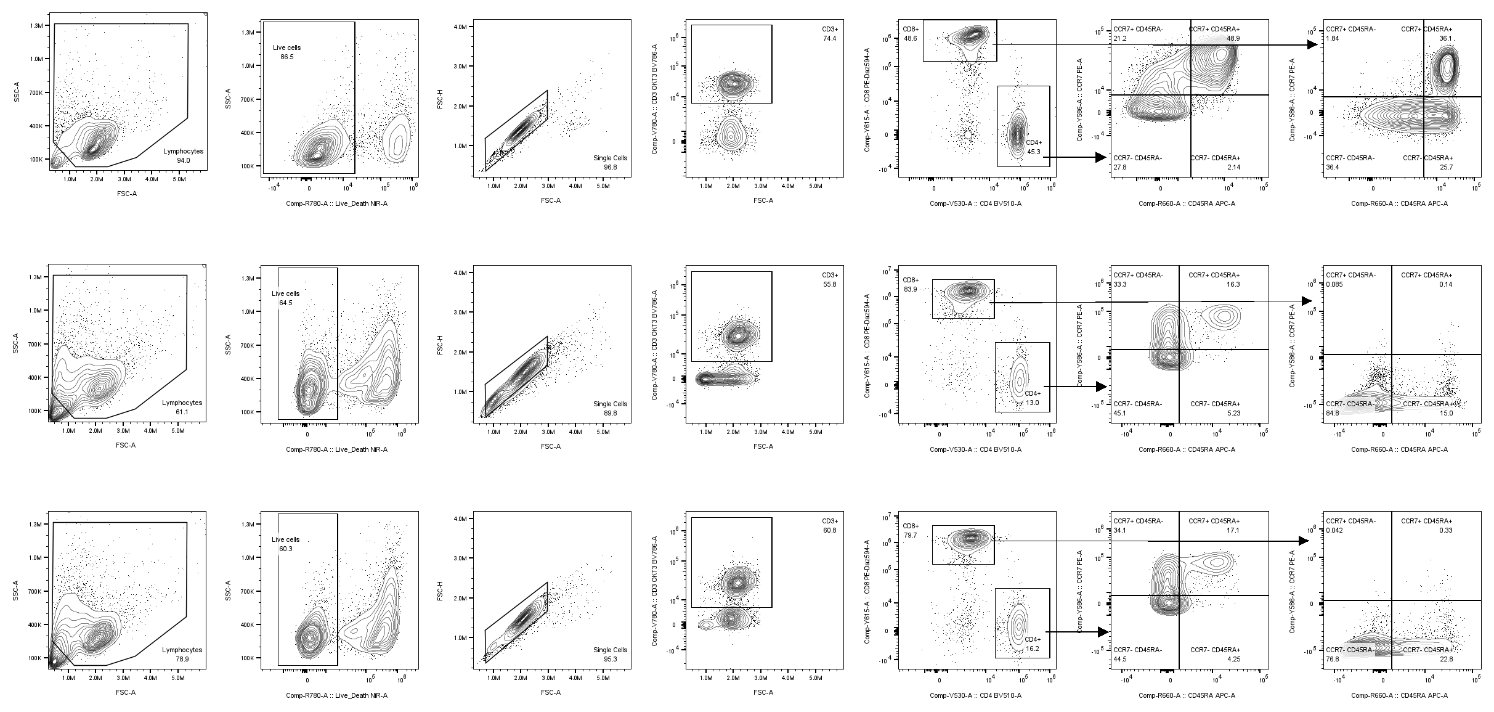 |
| --- |

**Supplementary Figure 1.** Gating strategy and flow plots from phenotype analysis of a representative healthy donor (DM_16) and patient samples (SC0169) at days +90 and +180 after HSCT.

|  | |
| --- | --- |
| Basal Respiration | A baseline measure of the rate of oxygen consumption by cells prior to addition of mitochondrial inhibitors such as Oligomycin. Basal respiration is an indicator of the oxygen consumption used to meet basic cellular ATP demand. Basal respiration is calculated as the difference in OCR before and after adding any mitochondrial modulators (Oligomycin or FCCP, dotted red line) and after adding antimycin A (dotted blue line) |
| Maximal respiration | Maximal rate of oxygen that can be consumed for oxidative phosphorylation. It is measured after the addition of FCCP which allow the free flow of protons across the inner mitochondrial membrane. Maximal respiration is calculated as the difference in OCR of cells after addition of FCCP and after treatment with antimycin A. |
| ATP turnover | ATP turnover describes the difference in OCR after inhibition of the ATP synthase with oligomycin. ATP turnover is therefore a relative to the ATP being produced by oxidative respiration. It is calculated as the difference in OCR before addition of mitochondrial modulators and after oligomycin. |
| Spare respiratory capacity | The theoretical extra capacity to produce ATP as a response to an increase in energetic demand. It is calculated as the difference between basal respiration and maximal respiration. |

**Supplementary Figure 2.** Representative plot of Seahorse measurements showing data from 2 patients (SC0104 & SC0147) at both +90 and +180 days after HSCT. Dotted lines represent timepoint of injections of Oligomycin or FCCP (red) and Antimycin A (blue).

#### Supplementary data

| **Patient ID** | **Days after HSCT** | **Basal respiration** | **ATP turnover** | **Maximum respiration** | **Spare respiratory capacity** |
| --- | --- | --- | --- | --- | --- |
| **SC0104** | +90 | 56.45 | 48.6214 | 77.72009 | 21.27447 |
|  | +180 | 55.77 | 45.70493 | 90.21403 | 34.44263 |
| **SC0118** | +90 | 23.93834 | 16.52213 | 158.51 | 134.5717 |
|  | +180 | 60.07945 | 50.02786 | 97.74151 | 37.66206 |
| **SC0138** | +90 | 49.13323 | 36.52842 | 126.5755 | 77.4423 |
|  | +180 | 6.230355 | 5.918814 | 11.05251 | 4.822155 |
| **SC0141** | +90 | 32.172 | 28.92007 | 122.1715 | 86.7427 |
|  | +180 | 35.53077 | 27.30907 | 78.29167 | 42.76091 |
| **SC0147** | +90 | 55.28089 | 45.20786 | 126.4552 | 71.17432 |
|  | +180 | 58.22465 | 47.68504 | 184.8312 | 126.6066 |
| **SC0151** | +90 | 51.04049 | 43.44868 | 180.881 | 129.8405 |
|  | +180 | 15.88301 | 15.01979 | 25.23689 | 9.353882 |
| **SC0152** | +90 | 53.99772 | 11.05365 | 105.0883 | 51.09053 |
|  | +180 | 9.904411 | 3.221364 | 41.7254 | 31.82099 |
| **SC0153** | +90 | 25.1864 | 0.585568 | 109.5609 | 84.37453 |
|  | +180 | 26.62426 | 6.778293 | 38.48118 | 42.53771 |
| **SC0166** | +90 |  |  |  |  |
|  | +180 | 13.52461 | 4.946383 | 33.37279 | 19.84818 |
| **SC0167** | +90 | 16.13358 | 4.933325 | 56.4459 | 40.31232 |
|  | +180 | 21.54235 | 3.339513 | 64.30915 | 42.7668 |
| **SC0168** | +90 | 44.9918 | 21.72733 | 95.35545 | 75.59415 |
|  | +180 | 36.56186 | 9.21078 | 97.24587 | 60.68401 |
| **SC0169** | +90 | 29.54057 | 6.14337 | 39.2029 | 65.34483 |
|  | +180 | 14.51934 | 4.321953 | 43.67696 | 32.41673 |
| **SC0171** | +90 | 3.236327 | 3.038433 | 8.812519 | 5.576192 |
|  | +180 | 13.21404 | 7.61135 | 30.29993 | 17.08589 |
| **SC0176** | +90 | 24.11165 | 19.11696 | 57.82571 | 33.71405 |
|  | +180 | 24.14712 | 20.78016 | 64.28894 | 40.14182 |
| **SC0182** | +90 | 23.29179 | 19.19644 | 89.45047 | 66.15868 |
|  | +180 | 12.88443 | 14.5014 | 53.91486 | 41.03043 |
| **SC0183** | +90 | 19.98018 | 15.63993 | 56.16835 | 36.18817 |
|  | +180 | 37.81507 | 31.31864 | 70.93384 | 33.11877 |
| **SC0186** | +90 | 30.01539 | 23.85743 | 98.4074 | 68.39201 |
|  | +180 | 21.37336 | 17.12844 | 79.98564 | 58.61229 |
| **SC0187** | +90 | 14.49653 | 12.39219 | 39.41573 | 24.9192 |
|  | +180 | 1.231314 | 0.76938 | 36.7515 | 35.52018 |
| **SC0192** | +90 | 25.57605 | 20.00705 | 142.0353 | 116.4593 |
|  | +180 | 30.81305 | 24.11697 | 141.4922 | 110.6792 |
| **SC0198** | +90 | 20.89783 | 14.06327 | 80.91254 | 60.01471 |
|  | +180 | 54.92788 | 39.35518 | 231.3764 | 176.4485 |
| **Healthy #1** |  | 41.65 | 26.61 | 119.06 | 77.41 |
| **Healthy #2** |  | 33.02 | 20.84 | 93.53 | 60.51 |
| **Healthy #3** |  | 37.44 | 23.95 | 113.74 | 76.30 |
| **Healthy #4** |  | 29.45 | 22.56 | 117.39 | 87.94 |
| **Healthy #5** |  | 44.45 | 29.69 | 154.62 | 110.17 |
| **Healthy #6** |  | 27.15 | 16.98 | 95.83 | 68.68 |
| **Healthy #7** |  | 32.46 | 5.30 | 148.12 | 115.66 |
| **Healthy #8** |  | 34.68 | 4.79 | 137.39 | 102.71 |
| **Healthy #9** |  | 42.20 | 11.11 | 154.22 | 112.02 |
| **Healthy #10** |  | 55.86 | 10.93 | 162.78 | 106.92 |
| **Healthy #11** |  | 68.55 | 13.48 | 173.45 | 104.90 |

**Supplementary data 1.** Table overview of the oxygen consumption rate (OCR, [pmol/min]) measured for HSCT patients at day +90 and +180 after HSCT. Basal respiration, ATP turnover and the maximal respiration are measured directly and the spare respiratory capacity is calculated as the difference between maximal- and basal respiration.
